# Clinical and biological markers for predicting ARDS and outcome in septic patients

**DOI:** 10.1038/s41598-021-02100-w

**Published:** 2021-11-22

**Authors:** Jesús Villar, Rubén Herrán-Monge, Elena González-Higueras, Miryam Prieto-González, Alfonso Ambrós, Aurelio Rodríguez-Pérez, Arturo Muriel-Bombín, Rosario Solano, Cristina Cuenca-Rubio, Anxela Vidal, Carlos Flores, Jesús M. González-Martín, M. Isabel García-Laorden, Genetics of Sepsis (GEN-SEP) Network, Ramón Adalia, Gerard Sánchez-Etayo, Alfonso Ambrós, Carmen Martín-Rodríguez, Elena González-Higueras, Rosario Solano, Laura Martínez-García, M. Isabel García-Laorden, Jesús Villar, Jesús M. González-Martín, Aurelio Rodríguez-Pérez, Ángel Becerra, Lucía Valencia, Demetrio Carriedo, Francisco Javier Díaz Domínguez, Anxela Vidal, José M. Añón, Pablo Millán, Domingo Martínez, Miryam Prieto-González, Cristina Cuenca-Rubio, Ana Isabel García-Sánchez, Braulio Álvarez-Martínez, Perfectino Fernández-Pérez, Efrén Otero-Alvarín, Carlos Flores, Gerardo Aguilar, Nasara Segura, Marina Soro, Rubén Herrán-Monge, Arturo Muriel-Bombín, Marta M. García-García, Concepción Tarancón, Teresa Álvarez

**Affiliations:** 1 CIBER de Enfermedades Respiratorias, Instituto de Salud Carlos III, 28029 Madrid, Spain; 2 Research Unit, Hospital Universitario de Gran Canaria Dr. Negrín, 35019 Las Palmas de Gran Canaria, Spain; 3 Keenan Research Center for Biomedical Sciences at the Li Ka Shing Knowledge Institute, St. Michael’s Hospital, Toronto, ON M5B 1W8 Canada; 4 Intensive Care Unit, Hospital Universitario Río Hortega, Gerencia Regional de Salud, SACYL, 47012 Valladolid, Spain; 5 GRECIA Group (Grupo de Estudio y Análisis en Cuidados Intensivos), Valladolid, Spain; 6 Group for Biomedical Research in Sepsis (BioSepsis), Instituto de Investigación Biomédica de Salamanca, (IBSAL), 37007 Salamanca, Spain; 7 Intensive Care Unit, Hospital Virgen de La Luz, 16002 Cuenca, Spain; 8 Intensive Care Unit, Complejo Asistencial Universitario de Palencia, 34005 Palencia, Spain; 9 Intensive Care Unit, Hospital General Universitario de Ciudad Real, 13005 Ciudad Real, Spain; 10 Department of Anesthesiology, Hospital Universitario de Gran Canaria Dr. Negrín, Universidad de Las Palmas de Gran Canaria, 35019 Las Palmas de Gran Canaria, Spain; 11 Intensive Care Unit, Hospital Universitario Fundación Jiménez Díaz, 28040 Madrid, Spain; 12 Research Unit, Hospital Universitario N. S. de Candelaria, 38010 Santa Cruz de Tenerife, Spain; 13 Genomics Division, Instituto Tecnológico y de Energías Renovables, 38600 Tenerife, Spain; 14 Department of Anesthesiology, Hospital Clinic de Barcelona, 08036 Barcelona, Spain; 15 Intensive Care Unit, Complejo Asistencial Universitario de León, 24001 León, Spain; 16 Intensive Care Unit, Hospital Universitario La Paz, 28046 Madrid, Spain; 17 Intensive Care Unit, Hospital Universitario Virgen de Arrixaca, 30120 Murcia, Spain; 18 Department of Clinical Analysis, Complejo Asistencial Universitario de Palencia, 34005 Palencia, Spain; 19 Intensive Care Unit, Hospital El Bierzo, 24404 Ponferrada, León, Spain; 20 Department of Anesthesia, Hospital Clínico Universitario, 46010 Valencia, Spain; 21 Intensive Care Unit, Hospital Virgen de la Concha, 49022 Zamora, Spain

**Keywords:** Biomarkers, Infectious diseases, Respiratory tract diseases

## Abstract

Sepsis is a common cause of acute respiratory distress syndrome (ARDS) associated with a high mortality. A panel of biomarkers (BMs) to identify septic patients at risk for developing ARDS, or at high risk of death, would be of interest for selecting patients for therapeutic trials, which could improve ARDS diagnosis and treatment, and survival chances in sepsis and ARDS. We measured nine protein BMs by ELISA in serum from 232 adult septic patients at diagnosis (152 required invasive mechanical ventilation and 72 had ARDS). A panel including the BMs RAGE, CXCL16 and Ang-2, plus PaO_2_/FiO_2_, was good in predicting ARDS (area under the curve = 0.88 in total septic patients). Best performing panels for ICU death are related to the presence of ARDS, need for invasive mechanical ventilation, and pulmonary/extrapulmonary origin of sepsis. In all cases, the use of BMs improved the prediction by clinical markers. Our study confirms the relevance of RAGE, Ang-2, IL-1RA and SP-D, and is novel supporting the inclusion of CXCL16, in BMs panels for predicting ARDS diagnosis and ARDS and sepsis outcome.

## Introduction

Sepsis is defined as an organ dysfunction resulting from a dysregulated host response to infection^1^. Sepsis is a common cause of acute respiratory distress syndrome (ARDS). Both syndromes are associated with a high mortality^2,3^. Sepsis and ARDS are highly heterogeneous, which hinders diagnosis and mortality estimation.

Having an easy to measure biomarker (BM) or a panel of BMs at the bedside, would be very useful for identifying patients at risk for ARDS, or at high probability of fatal outcome. The use of BMs to implement understanding of how ARDS or sepsis evolve via application of agonist or antagonist of certain BM would have an impact of personalized treatment for increasing survival in sepsis and ARDS. Although at the present, there are no therapies available that, given early knowledge of serum/plasma levels of any BM, would prevent or mitigate the development of ARDS or its associated mortality, there is a hope that early stratification of patients based on the levels of selected BMs at the time of sepsis/ARDS onset, or within the first 24 h, could represent a novel strategy for early stratification of sepsis/ARDS into prognostic categories and for selecting patients for therapeutic trials.

Candidate protein BMs are selected based on their biological roles in the disease process. In the case of ARDS, markers of endothelial and epithelial injury, inflammation, coagulation, fibrosis, and apoptosis, have been examined^4–6^. Terpstra et al.^7^ performed a meta-analysis and provided a ranking of individual BMs associated with ARDS diagnosis and outcome. Another group has published several reports on panels of BMs, alone or in combination with clinical variables. They have reported a panel of 7 BMs with elevated capacity to discern between patients with and without acute lung injury in critically ill patients with traumatic injuries^8^, and a panel of 5 BMs able to predict ARDS in patients with severe sepsis^9^. They have also found that the prediction of ARDS outcome improved when combining BMs and clinical predictors^10^. More recently, they have validated a model combining two BMs and a clinical variable to predict hospital mortality in ARDS patients^11^.

We aimed to determine a small panel of biological and clinical markers for an early identification of septic patients at risk for developing ARDS, and with higher probability of fatal outcome. We sought that these panels would help to optimize personalized treatment in sepsis and ARDS. For this purpose, in septic patients with and without ARDS, we measured serum levels of BMs identifiers of diverse pathophysiological changes during the progression of the disease: receptor for advanced glycation end-products (RAGE) and surfactant protein (SP)-D as indicative of alveolar epithelium damage; angiopoietin (Ang)-2 and intercellular adhesion molecule (ICAM)-1 as markers of vascular endothelium damage; interleukin (IL)-18 and IL-1 receptor antagonist (IL-1RA) as mediators in the inflammatory response; and plasminogen activator inhibitor-1 (PAI-1) as indicative of fibrinolysis. Based on previous studies, we also measured the proteins amphiregulin (AREG)^12^ and chemokine (C-X-C motif) ligand 16 (CXCL16)^13^.

## Methods

### Study setting

Clinical data and blood samples were collected between 2012 and 2020 as part of the GEN-SEP study, a national, multicenter, observational study conducted in a network of Spanish Intensive Care Units (ICUs). The purpose of the present study was to investigate a series of BMs for a better prediction of ARDS development and mortality in septic patients. The study was approved by the local ethics committee of all participant hospitals (approved by the Ethics Committee for Clinical research of Hospital Universitario Río Hortega [2011-3-3] and by the Research Ethics Committee/Committee of Ethics of Research with Medicines of Hospital Universitario de Gran Canaria Dr. Negrín [2019-031-1], and adopted by all participating centers, as required by Spanish legislation), and conducted in accordance to the Spanish legislation and the Declaration of Helsinki. Written informed consent was obtained from all patients or their relatives. Samples are stored at the Research Unit of the Hospital Universitario de Gran Canaria Dr. Negrín in a collection registered in the National Registry of Biobanks (C.0005149).

### Study population and data collection

Two hundred thirty-two adult patients (87.6% Caucasian) who fulfilled sepsis criteria^1^ shortly before or within the first 24 h of ICU admission were studied. A total of 152 patients required invasive mechanical ventilation (IMV), and 72 of them met ARDS criteria. An overview of the main groups of patients is shown in Supplementary Fig. S1 online. Sepsis was defined by Sepsis-3 criteria^1^ and ARDS by the Berlin definition^14^. Infection was considered when microbiologically documented according to the Center for Disease Control and Prevention definitions, or when clinical suspicion with evidence was present. Patients with a terminal disease, chronic obstructive pulmonary disease or congestive heart failure were excluded.

Clinical and demographical data for the diagnosis of sepsis and ARDS, and for assessing disease severity, were prospectively collected from all patients. Acute Physiology and Chronic Health Evaluation II (APACHE II) score^15^ and Sequential Organ Failure Assessment (SOFA) score^16^ were recorded at diagnosis. Number of organs/systems with dysfunction or failure was calculated considering dysfunction/failure of each organ system as an increase of 1 or greater on its SOFA score. Patients were followed up until hospital discharge or death. Duration of IMV, length of ICU and hospital stay, and ICU and hospital mortality, were also recorded.

### Samples and assays

Serum samples were obtained from patients at the time of study inclusion (within the first 24 h after diagnosis). Sodium citrate plasma samples were obtained from an additional group of 60 septic patients on IMV (29 of them with ARDS). Serum and plasma samples were kept at -80ºC until use. Levels of RAGE, PAI-1, SP-D, IL-18, Ang-2, ICAM-1, AREG, IL-1RA and CXCL16, were measured by ELISA using DuoSet ELISA kits and DuoSet Ancillary Reagent Kit2 (R&D Systems, Abingdon, UK) following the manufacturer's protocol. Samples were measured in duplicate. The lower limits of detection of the assays were 62.5, 0.31, 156.0, 11.7, 93.8, 31.25, 15.6, 39.1 and 15.6 pg/mL respectively.

### Statistical analysis

Clinical and demographic variables are reported as frequency and percentage for categorical data, mean and standard deviation for continuous parametric data or median with quartiles 1 and 3 (Q1-Q3) for continuous non parametric data. Normal distribution of continuous variables was tested by Kolmogorov–Smirnov test. For BMs levels, values below the limit of detection were imputed as half the lower detection limit for each biomarker. Categorical data were compared with Chi-squared test or Fischer’s exact test when needed, continuous non parametric variables were compared using Mann–Whitney U test for two independent groups. For comparison of single BMs, raw data were used. For any other analysis, BMs values underwent logarithmic transformation to achieve approximate normality. Univariable logistic regression (for single BMs and clinical variables) and backward stepwise multivariate logistic regression (for grouped BMs and BMs plus clinical variables) were performed. Subsequently, the most optimal variables and panels of variables were selected based on its predictive performance according to the computed receiver operating characteristics (ROC) curves and their area under the curve (AUC), which is reported with a 95% confidence interval (CI). For univariable and multivariable analysis with 28-day ICU survival as dependent variable, Cox regression model was used. Then, the optimal cut-off point value of the ROC curve for prediction of 28-day ICU mortality of the single BMs and clinical variables of interest were calculated. One point was assigned to each of these variables in individuals with values higher than the cut-off point, and a final score was calculated by the sum of the variables of the panel of interest. The cut-off value for the score of each panel was calculated, and individuals were classified based on having a score higher or lower than the aforementioned value. Next, survival rates were estimated by the Kaplan–Meier method, and their comparison was performed with the log-rank test. For all analysis, SPSS Statistical Package version 15.0 (SPSS Inc., Chicago, IL, USA) was used. For all comparisons, a two-tailed *P* value < 0.05 was considered significant.

## Results

### Patient characteristics

Clinical and demographic characteristics of main groups are shown in Table 1. There were significant differences between sepsis patients with or without IMV, and with or without ARDS. Patients on IMV had higher severity scores (APACHE II score: *P* = 0.006, SOFA score: *P* = 2*10^−5^), higher ICU and hospital length of stay (*P* = 1.9*10^−22^ and *P* = 4.0*10^−8^ respectively), and higher mortality (*P* = 9*10^−6^) than patients without IMV. In patients with IMV, those with ARDS had increased number of days on IMV (*P* = 0.022) and higher mortality (*P* = 0.002) than patients without ARDS (Table 1).

**Table 1:** Clinical and demographical characteristics of the main study groups.

Characteristic	Total sepsis (N = 232)	Sepsis without IMV (N = 81)	Sepsis with IMV (N = 151)	Non-ARDS sepsis with IMV (N = 79)	ARDS sepsis with IMV (N = 72)
Age, years Mean ± STD	63.7 ± 15.2	66.7 ± 14.4	62.1 ± 15.4*	64.2 ± 16.5	59.7 ± 13.8
Gender, male N (%)	135 (58.2)	45 (55.6)	90 (59.6)	48 (60.8)	42 (58.3)
**Cause of sepsis N (%)**					
Pulmonary	86 (37.1)	11 (13.6)	75 (49.7)***^a^	26 (32.9)	49 (68.1)^###a^
Extrapulmonary	141 (60.8)	67 (82.7)	74 (49.0)	52 (65.8)	22 (30.6)
Unknown	5 (2.2)	3 (3.7)	2 (1.3)	1 (1.3)	1 (1.4)
APACHE II score median (Q1–Q3)	17 (13–23)	16 (11.5–20.5)	19 (14–24)**	19 (14–24)	18 (14–24)
SOFA score median (Q1–Q3)	8 (6–10)	7 (4–8)	8 (6–11)***	8 (6–11)	9 (7–10.8)
Cardiovascular system median (Q1–Q3)	4 (3–4)	3 (2–4)	4 (3–4)**	4 (3–4)	4 (3–4)
Respiratory system median (Q1–Q3)	2 (1–3)	1 (0–2)	3 (2–3)***	2 (2–3)	3 (3–4)^###^
Hepatic system median (Q1–Q3)	0 (0–0)	0 (0–0.5)	0 (0–0)	0 (0–0)	0 (0–0)
Renal system median (Q1–Q3)	1 (0–2)	1 (0–2)	1 (0–2)	1 (0–2)	0 (0–2)
Neurological system median (Q1–Q3)	0 (0–1)	0 (0–0)	0 (0–1)*	0 (0–1)	0 (0–1)
Coagulation system median (Q1–Q3)	0 (0–1)	0 (0–1)	0 (0–1)	0 (0–1)	0 (0–1)
NOA median (Q1–Q3)	3 (2–4)	3 (2–4)	3 (2–4)*	3 (2–5)	3 (3–4)
NEOA median (Q1–Q3)	2 (1–3)	2 (2–3)	2 (1–3)	3 (1–4)	2 (2–3)
PaO_2_/FiO_2_ median (Q1–Q3)	210 (133.3–341.7)	350 (234.8–500)	179.2 (123–249)***	214 (151.7–294.3)	135 (106.5–184)^###^
Days on IMV median (Q1–Q3)	5 (0–12)	0	10 (5–20)	8.5 (4–15)	11.5 (6–24.5)^#^
ICU days median (Q1–Q3)	8 (4–18)	3 (2–5)	14 (7–29)***	13 (7–27)	16 (10–34)
Hospital days median (Q1–Q3)	24 (14.3–46.5)	15 (10–27.5)	31 (18–51)***	31 (19–51)	31 (17.3–52.5)
ICU mortality N (%)	37 (15.9)	2 (2.5)	35 (23.2)***	10 (12.7)	25 (34.7)^##^

*IMV* invasive mechanical ventilation, *ARDS* acute respiratory distress syndrome, *APACHE II* acute physiology and chronic health evaluation II, *SOFA* sequential organ failure assessment, *NOA* number of total organs affected, *NEOA* number of extrapulmonary organs affected, *ICU* intensive care unit.

*P < 0.05, **P < 0.01 and ***P < 0.001 for the comparison of sepsis with and without IMV.

^#^*P* < 0.05, ^##^*P* < 0.01 and ^###^*P* < 0.001 for the comparison of ARDS sepsis with IMV and non-ARDS sepsis with IMV.

^a^Pulmonary versus extrapulmonary sepsis.

### Biomarkers levels

Serum levels of BMs at sepsis diagnosis are shown in Table 2. Patients who required IMV presented significantly increased levels of RAGE, PAI-1, SP-D, CXCL16 and AREG (*P* = 2*10^−6^, *P* = 0.012, *P* = 0.009, *P* = 7*10^−9^ and *P* = 0.003 respectively), and significantly decreased levels of Ang-2 (*P* = 0.033) than patients without IMV (Table 2). The group of patients with pulmonary sepsis exhibited higher levels of RAGE (*P* = 4*10^−7^) and SP-D (*P* = 1*10^−5^) and lower levels of PAI-1, Ang-2 and ICAM-1 (*P* = 0.016, *P* = 1*10^−6^ and *P* = 0.036 respectively) compared to patients with extrapulmonary sepsis (Table 2). Additional data on serum samples and comparison to plasma levels are presented in Supplementary Table S1 online.

**Table 2:** Biomarkers levels at sepsis diagnosis.

Biomarker	Total sepsis (N = 232)	Sepsis without IMV (N = 81)	Sepsis with IMV (N = 151)	Extrapulmonary sepsis (N = 141)	Pulmonary sepsis (N = 86)
RAGE (pg/mL)	1019.66 (563.37–2066.59)	750.19 (440.10–1181.38)	1371.12*** (645.05–2581.35)	828 (455.34–1408.46)	1825..38^###^ (848.91–3089.29)
PAI-1 (pg/mL)	73.12 (43.08–140.18)	58.80 (36.00–108.94)	82.93* (46.50–202.72)	84.87 (47.47–165.21)	55.35^#^ (38.06–112.48)
SP-D (ng/mL)	5.80 (2.54–11.39)	4.17 (1.79–8.81)	6.73** (2.89–13.47)	4.46 (1.81–8.85)	8.03^###^ (4.39–16.87)
IL-18 (pg/mL)	543.17 (300.71–1056.15)	521.28 (297.19–920.67)	559.11 (303.20–1153.49)	505.52 (297.19–985.91)	567.51 (302.01–1000.91)
Ang-2 (pg/mL)	4467.88 (2169.10–8352.27)	5302.58 (3070.03–10,110.21)	3851.28* (1906.46–8105.40)	5524.24 (2885.51–10,360.47)	2914.04^###^ (1325.90–5616.83)
ICAM-1 (ng/mL)	356.71 (261.70–518.09)	324.27 (255.99–516.93)	383.21 (261.18–525.07)	372.55 (271.29–573.70)	325.62^#^ (233.10–489.05)
CXCL16 (pg/mL)	4255.27 (2771.32–6361.93)	2985.59 (2269.12–4241.76)	5020.01*** (3400.91–7133.91)	4229.28 (2,857,035–6040.27)	4286.74 (2620.53–6474.89)
AREG (pg/mL)	38.05 (20.20–82.94)	29.87 (7.80–64.97)	45.81** (26.55–101.06)	38.98 (23.02–79.81)	37.21 (16.11–98.12)
IL-1RA (pg/mL)	603.74 (19.55–3248.49)	232.32 (19.55–232.32)	820.32 (19.55–10,325.96)	816.98 (19.55–4950.81)	239.92 (19.55–1932.36)

Concentrations are given as median (Q1–Q3).

N is the number of samples in each group.

*IMV* invasive mechanical ventilation.

*P < 0.05, **P < 0.01 and ***P < 0.001 for the comparison of sepsis with and without IMV.

^#^*P* < 0.05 and ^###^*P* < 0.001 for the comparison of extrapulmonary and pulmonary sepsis.

### Biomarkers and clinical variables at ARDS diagnosis

**Figure 1:**
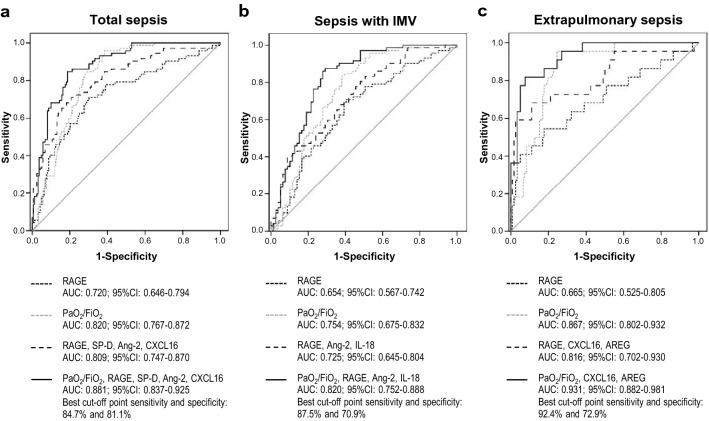
Predictive value of biomarkers and clinical variables in ARDS diagnosis in septic patients. Panels represent ROC curve analysis comparing predictive value of the best performing BM, clinical variable, BMs panel and final panel combining BMs and clinical variable in patients with (**a**) sepsis, (**b**) sepsis requiring IMV, and (**c**) extrapulmonary sepsis. *ARDS* acute respiratory distress syndrome, *BM* biomarker, *IMV* invasive mechanical ventilation.

Serum levels of individual BMs and values of clinical variables at diagnosis in septic patients with and without ARDS are shown in Supplementary Table S2. Among BMs, RAGE showed the highest AUC value (Fig. 1a). After performing a logistic regression model and backward stepwise multivariate logistic regression, selected BMs were RAGE, SP-D, Ang-2 and CXCL16. PaO_2_/FiO_2_ was the best clinical predictor of ARDS. A model including selected BMs and PaO_2_/FiO_2_ showed a better discrimination for ARDS diagnosis than BMs or clinical variables alone (Fig. 1a). When comparing non-ARDS septic patients who needed IMV to septic patients with ARDS, RAGE, SP-D and Ang-2 were significantly different (*P* = 0.001, *P* = 0.014 and *P* = 0.003 respectively) (Supplementary Table S2 online), and RAGE had the best predictive value for ARDS (Fig. 1b). The panel including RAGE, Ang-2 and IL-18 was selected using backward stepwise multivariate logistic regression. Discrimination for ARDS diagnosis was better when using the model including those three BMs and the PaO_2_/FiO_2_ than when using the BMs or the PaO_2_/FiO_2_ alone (Fig. 1b). In patients with extrapulmonary sepsis, diverse BMs and clinical variables differed between patients with or without ARDS (Supplementary Table S2 online), The best BM panel, which included RAGE, CXCL16 and AREG, had a worse predictive value than the best clinical variable (PaO_2_/FiO_2_) (Fig. 1c). However, the predictive value of the model was excellent when including those three BMs and the PaO_2_/FiO_2_ (Fig. 1c). Similar results were observed in patients with extrapulmonary sepsis who required IMV (N = 75): AUC of the panel RAGE, CXCL16, AREG plus PaO_2_/FiO_2_ was 0.898 (95% CI 0.825–0.970). In patients with pulmonary sepsis, neither BMs nor clinical variables were good predictors for ARDS (data not shown).

Based on these results, and with the aim of having a unique minimal panel useful for any of the situations mentioned above, we tested a panel with three BMs (RAGE, CXCL16 and Ang-2) and a clinical marker (PaO_2_/FiO_2_) to predict ARDS in all groups of patients. The performance of this panel was: for the cohort of septic patients AUC: 0.877, 95%CI: 0.833–0.921; for septic patients on IMV, AUC: 0.805, 95%CI: 0.735–0.876; for patients with extrapulmonary sepsis, AUC: 0.916, 95%CI: 0.861–0.971; for patients with extrapulmonary sepsis on IMV, AUC: 0.881, 95%CI: 0.799–0.964.

### Predictive value of biomarkers and clinical variables in ICU mortality

**Figure 2:**
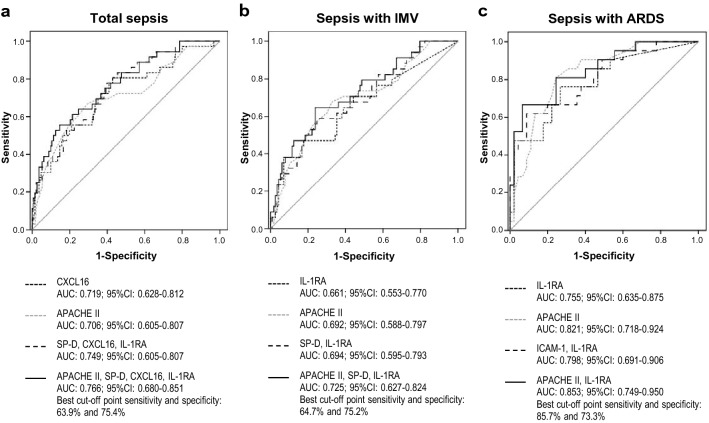
Predictive value of biomarkers and clinical variables on ICU mortality of septic patients. Panels represent ROC curve analysis comparing predictive value of the best performing BM, clinical variable, BMs panel and final panel combining BMs and clinical variable in patients with (**a**) sepsis, (**b**) sepsis requiring IMV, and (**c**) sepsis with ARDS. *ICU* intensive care unit, *BM* biomarker, *IMV* invasive mechanical ventilation, *ARDS* acute respiratory distress syndrome.

Some BMs were significantly elevated in septic patients who died in ICU when compared to patients alive at ICU discharge (Supplementary Table S3 online). The best BM predictor was CXCL16, and a backward stepwise multivariate logistic regression yielded a reduced model including SP-D, CXCL16 and IL-1RA (Fig. 2a). APACHE II score was the best clinical predictor, and in combination with the selected three BMs showed an AUC of 0.766 (Fig. 2a). Similar results were found for individual BMs when considering only septic patients who required IMV (Supplementary Table S3 online), and a model including SP-D and IL-1RA was obtained (Fig. 2b). The best predictive panel included these two BMs and APACHE II score (Fig. 2b). When examining the prediction of ICU death in ARDS patients, the values of some BMs and clinical variables were higher in non-survivors (Supplementary Table S3 online). While the best predictive BM panel included IL-1RA and ICAM-1, the best final predictor model was IL-1RA plus APACHE II score (Fig. 2c).

Serum BMs and clinical variables in ICU survivors and non-survivors in patients with extrapulmonary and pulmonary sepsis are shown in Supplementary Table 3 online. The best single BM and clinical variable predictor, the selected BMs panel, and the best final predictive model in patients with extrapulmonary sepsis (9.86% of mortality) and patients with pulmonary sepsis (24.42% of mortality), are shown in Supplementary Fig. S2 online. When analyzing patients on IMV, the best predictive model for extrapulmonary sepsis included APACHE II score, IL-18, Ang-2 and IL-1RA (AUC: 0.835, 95%CI: 0.716–0.954), and for pulmonary sepsis included APACHE II score, Ang-2 and ICAM-1 (AUC: 0.784, 95%CI: 0.666–0.902).

### Predictive value of biomarkers and clinical variables on cumulative ICU survival

**Figure 3:**
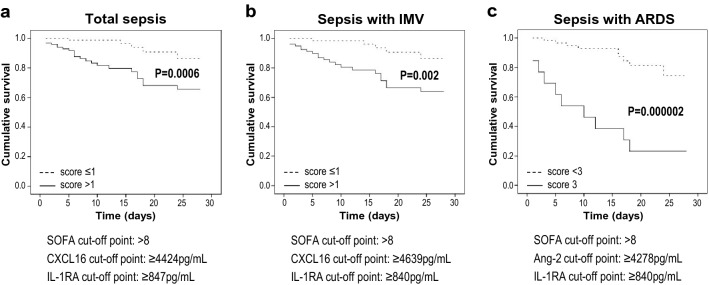
Predictive value of biomarkers and clinical variables on cumulative ICU survival in septic patients. Panels represent Kaplan–Meier survival curves for low and high scores of the best performing panels in patients with (**a**) sepsis, (**b**) sepsis requiring IMV, and (**c**) sepsis with ARDS. The BMs and clinical variable that integrate the best performing panels are presented under the plots, including the cut-off values used for the scoring. Dotted lines represent the groups of patients with low total scores, and solid lines those with high total scores. *ICU* intensive care unit, *BM* biomarker, *IMV* invasive mechanical ventilation, *ARDS* acute respiratory distress syndrome.

ICU survival at 28-day was associated with highly significant values of IL-1RA serum levels (*P* = 0.00006, HR 1.31, 95% CI 1.15–1.50) and SOFA score (*P* = 0.001, HR 1.18, 95% CI 1.07–1.30) in septic patients. The best performing variables when assessing cumulative 28-day ICU survival in individuals with values higher than the cut-off point, were CXCL16, IL-1RA and SOFA score (log-rank test *P* = 0.007, *P* = 0.002 and *P* = 0.003 respectively). When combining individual scoring of these variables, patients with a final score > 1 had higher 28-day cumulative ICU survival (Fig. 3a). The same variables were the best when studying septic patients who required IMV (Fig. 3b). In ARDS patients, those with a score of 3 for the panel Ang-2, IL-1RA and SOFA, had a significantly higher 28-day cumulative ICU mortality than the low-score group (Fig. 3c).

In patients with extrapulmonary sepsis, the best performing variables were PAI-1 and SOFA score: patients had higher 28-day cumulative ICU mortality when the two selected variables were above the cut-off point (score = 2; *P* = 0.000006) (Supplementary Fig. S3 online). The optimal panel for patients with pulmonary sepsis included ICAM-1, AREG and number of extrapulmonary organs affected, showing the high-score group (> 1) a significantly higher 28-day cumulative ICU survival than the low-score group (*P* = 0.0002) (Supplementary Fig. S3 online). In patients on IMV, the best predictive model for extrapulmonary sepsis included SOFA score, IL-18, and IL-1RA (log-rank *P* = 0.002), whereas for pulmonary sepsis included PaO_2_/FiO_2_, ICAM-1 and IL-1RA (log-rank *P* = 0.0002).

## Discussion

We have analyzed serum levels of nine candidate BMs representative of different pathophysiological disease-related changes during sepsis development: some of them are well known markers, but others are rarely studied or novel in this context. We found a panel that provided a good ARDS prediction in patients with sepsis. Best performing panels for ICU death and survival prediction relate to characteristics such as the presence of ARDS, need for IMV, and pulmonary or extrapulmonary origin of sepsis. In all cases, the use of BMs improves the prediction by clinical markers.

Candidate BMs of ARDS susceptibility have been previously described, although none has been universally accepted^5,6^. This could be due to the heterogeneity of ARDS etiology and phenotype, or because BMs levels are altered in septic patients as a result of activation of infectious and inflammatory processes. This would make the use of a panel of combined markers more suitable. We found association of ARDS with some individual serum BMs that had been found increased in plasma previously, as the markers of epithelial injury RAGE and SP-D, and the endothelial injury marker Ang-2^9,17–19^. Surprisingly, we found a decrease in Ang-2 serum levels in ARDS. We confirmed this decrease in plasma (data not shown), although we have no explanation for the difference from previous studies.

The combination of BMs has better performance than individual BMs, as reported in several studies combining diverse BMs to stablish panels with high discrimination of critical patients in risk of developing acute lung injury or ARDS^8,9,20^. In our study, we defined a narrow panel with good ARDS prediction for any of the analyzed sepsis groups. This panel includes the two widely associated BMs RAGE and Ang-2^8,9,20^, and the not-so-well studied CXCL16. The usefulness of RAGE and Ang-2 in ARDS prediction is understandable based on their biological roles in the disease process. Activation of RAGE, which is abundantly expressed on alveolar type 1 epithelial cells, has a role in cell signaling and propagation of the proinflammatory response^4–6^. Ang-2 is an endothelial growth factor which decreases endothelial junction integrity and, hence, enhances vascular leak and promotes vascular regression and cell death^4,5^. The role of CXCL16 in lung is poorly documented. In addition to its participation as a chemokine, a hint of possible processes involved comes from a study on human lung fibroblasts, reporting that CXCL16 facilitates fibrosis by enhancing proliferation, migration and collagen production^21^, and from a study associating CXCL16 with ARDS in patients with severe pneumonia^13^. Finally, when the PaO_2_/FiO_2_ ratio was combined with the 3-BM panel, we built a model with good predictive value, superior to any single clinical variable.

Since sepsis is a very heterogeneous syndrome with a wide range of organ dysfunction and clinical manifestations, it is extremely difficult to find a unique panel for prediction of death. The best ICU mortality predictor panels for all septic patients, for patients on IMV, for ARDS patients, and for those with pulmonary sepsis, included the APACHE II score as a clinical variable, and the BM IL-1RA. In the case of ARDS, these two markers set up the best mortality predictor panel. Multiple BMs have been studied to predict mortality from ARDS, but none is widely used in clinical practice due to the lack of reproducibility for most of them^7,19^. Panels of BMs have also been studied for prediction of death in ARDS, with improved performance when combined with clinical markers^10,11,22^. IL-1RA, an anti-inflammatory cytokine released during acute inflammatory responses, was not included in those studies. However, a recent report identified a 6-BM panel predicting mortality in ARDS, where IL-1RA was included^23^. In addition, Potjo et al.^24^ found that IL-1RA predicted mortality in sepsis with reasonable accuracy. The best predictor models for all septic patients and patients on IMV also included SP-D, while CXCL16 was included in the final panel for the entire cohort of septic patients. The reason for the absence of SP-D in best panels for patients with ARDS or with pulmonary sepsis is not clear, since SP-D is a marker of lung epithelial injury. A plausible explanation could be related to the smaller sample size of these groups. Similarly, the panel for septic patients with extrapulmonary origin could be due to the low number of deaths in that group.

The 28-day sepsis cumulative survival was predicted by panels including IL-1RA and SOFA score in all septic patients, in patients on IMV and in those with ARDS. The best panel for all septic patients and for septic patients on IMV was completed with the inclusion of CXCL16. The involvement of CXCL16 could be explained on the bases of its proposed role in fibrosis^21^. Why the best panel for ARDS included Ang-2 instead of CXCL16, is not clear. It is important to keep in mind that we are selecting the best performing and narrow panels from different options. When referring to cumulative survival in patients with extrapulmonary sepsis, it is worth noting that the small number of deaths in that group occurred earlier than in the other groups. This could be a modifying factor of the dead-related BMs present at sepsis diagnosis in this group. As a result, our findings highlight the complexity of obtaining a dead-predicting model valid for each septic condition. It is clear that IL-1RA is consistent in most groups, and CXCL16 seems to be also relevant. The latter has been associated with death in cardiovascular disease^25,26^ but, to our knowledge, our study is novel in relating it to sepsis mortality.

Most clinical studies measuring blood protein BMs in sepsis and in ARDS have been performed using plasma, while our study has been performed using serum. To evaluate differences in BMs levels between serum and plasma, we measured the BMs in plasma samples from an additional group of septic patients on IMV. This group had the same proportion of ARDS patients than the group of septic patients on IMV in which we analyzed serum concentration. PAI-1, IL-18 and Ang-2 had significant differences between serum and plasma levels, suggesting that these BMs cannot be measured interchangeably in both types of samples for comparison or for defining cut-off values. As a result, the BM panels described in our study should be validated in plasma in future studies.

We acknowledge several limitations in our study. First, a larger sample size would be desirable to increase statistical power, especially for mortality analysis. Second, it is plausible that other combination of BMs could generate a panel with a better predictive value, both for ARDS prediction and for ICU mortality. Third, further analysis would be necessary to validate our findings in serum and to confirm our panels in plasma samples. Due to the small sample size of our plasma group, we did not use it to replicate the study. Ideally, we should have done the comparison of BMs levels in plasma vs serum from the same individuals but, unfortunately, that was not possible. However, according to the homogeneous characteristics of both groups of patients, we should not expect relevant differences.

In conclusion, our study confirms the important contribution of RAGE and Ang-2 and supports the novel inclusion of CXCL16, together with the clinical marker PaO_2_/FiO_2_, to build a panel with good ARDS prediction among septic patients. It also confirms the relevance of several previously associated BMs, such as IL-1RA, SP-D and Ang-2, for panels predicting mortality or cumulative survival in septic patients, being APACHE II and SOFA scores the respective clinical variables of interest, while it is novel showing the utility of including CXCL16 in the panel. The panels for prediction of mortality and cumulative survival present some variability in their composition depending on the subgroups of septic patients. Further studies are necessary to validate the use of these panels in plasma samples.

## Supplementary Information

Supplementary Information.

## Data Availability

All data generated or analysed during this study are included in this published article and its additional files, or are available from the corresponding author on reasonable request.
