# Supplementary material for: Clinical and biological markers for predicting ARDS and outcome in septic patients
Source: Sci Rep. 2021 Nov 22;11:22702. doi: 10.1038/s41598-021-02100-w (PMC8608812; doi:10.1038/s41598-021-02100-w)
Supplement: Supplementary file 1 — Supplementary Information. [file 41598_2021_2100_MOESM1_ESM.pdf]

SUPPLEMENTARY MATERIAL

Clinical and biological markers for predicting ARDS and outcome in septic patients

Jesús Villar[1, 2 ,3], Rubén Herrán-Monge[4, 5, 6], Elena González-Higueras[7], Miryam Prieto-González[8], Alfonso Ambrós[9], Aurelio Rodríguez-Pérez[10], Arturo Muriel-Bombín[4, 5, 6], Rosario Solano[7], Cristina Cuenca-Rubio[8], Anxela Vidal[11], Carlos Flores[1, 12, 13], Jesús M. González-Martín[2], M. Isabel García-Laorden[1, 2] and Genetics of Sepsis (GEN-SEP) Network

[1]CIBER de Enfermedades Respiratorias, Instituto de Salud Carlos III, Madrid, Spain, [2]Research Unit, Hospital Universitario de Gran Canaria Dr. Negrín, Las Palmas de Gran Canaria, Spain, [3] Keenan Research Center for Biomedical Sciences at the Li Ka Shing Knowledge Institute, St. Michael's Hospital, Toronto, Canada, [4]Intensive Care Unit, Hospital Universitario Río Hortega, Gerencia Regional de Salud, SACYL, Valladolid, Spain. [5]GRECIA group (Grupo de Estudio y Análisis en Cuidados Intensivos). [6]Group for Biomedical Research in Sepsis (BioSepsis), Instituto de Investigación Biomédica de Salamanca, (IBSAL), Salamanca, Spain. [7]Intensive Care Unit, Hospital Virgen de La Luz, Cuenca, Spain, [8]Intensive Care Unit, Complejo Asistencial Universitario de Palencia, Palencia, Spain, [9]Intensive Care Unit, Hospital General Universitario de Ciudad Real, Ciudad Real, Spain, [10]Department of Anesthesiology, Hospital Universitario de Gran Canaria Dr. Negrín, Universidad de Las Palmas de Gran Canaria, Las Palmas de Gran Canaria, Spain, [11]Intensive Care Unit, Hospital Universitario Fundación Jiménez Díaz, Madrid, Spain, [12]Research Unit, Hospital Universitario N. S. de Candelaria, Santa Cruz de Tenerife, Spain, [13]Genomics Division, Instituto Tecnológico y de Energías Renovables, Tenerife, Spain.

SUPPLEMENTARY FIGURES

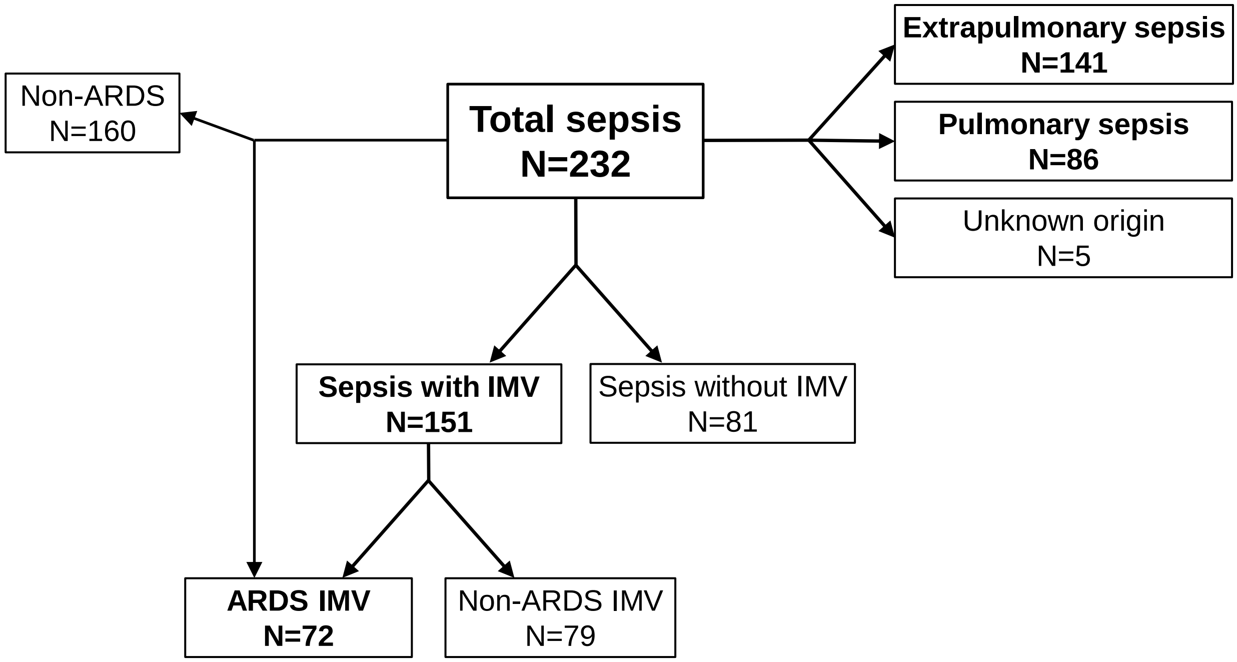

Supplementary Figure S1. Overview of the main subgroups of patients analyzed.

ARDS: Acute respiratory distress syndrome. IMV: Invasive mechanical ventilation.

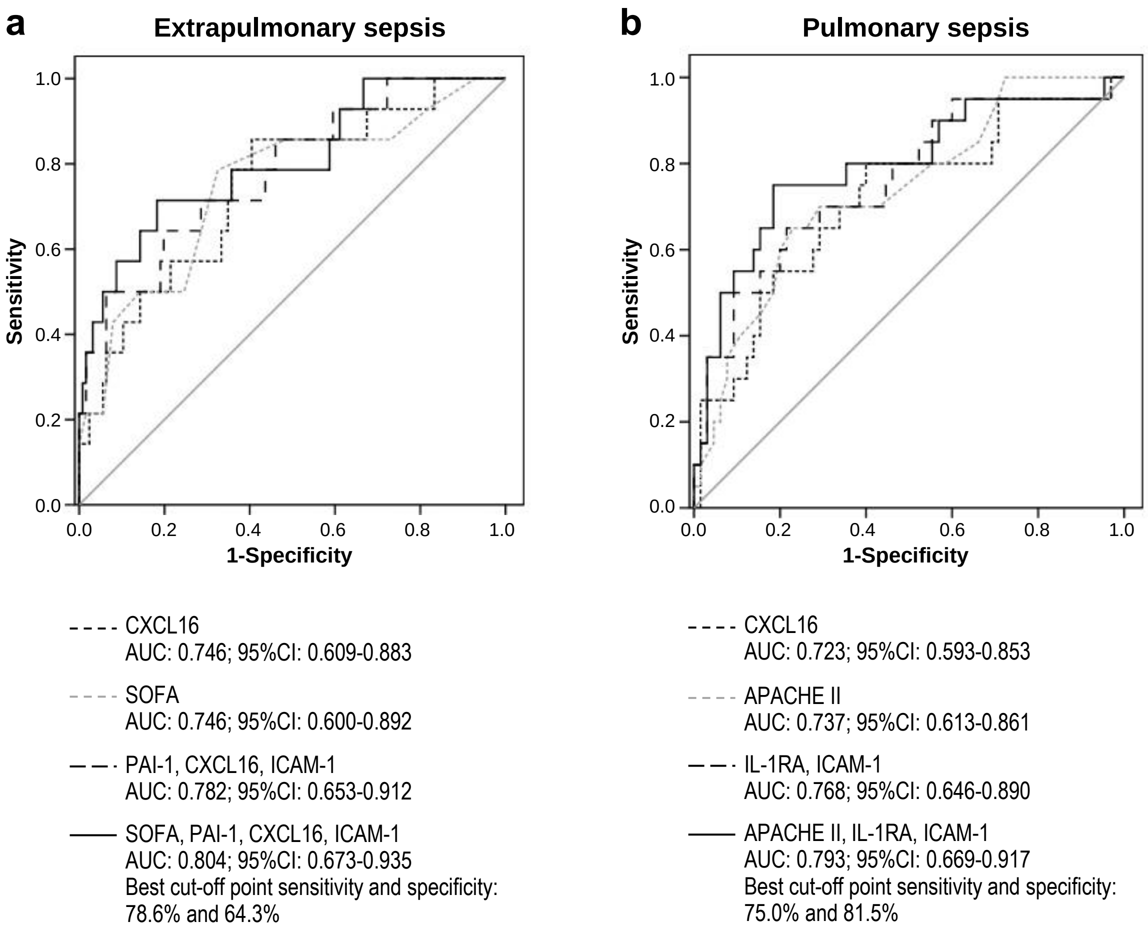

Supplementary Figure S2. Predictive value of biomarkers and clinical variables on ICU mortality of septic patients.

Panels represent ROC curve analysis comparing predictive value of the best performing BM, clinical variable, BMs panel and final panel combining BMs and clinical variable in patients with (a) extrapulmonary sepsis, and (b) pulmonary sepsis. ICU: Intensive Care Unit. BM: biomarker.

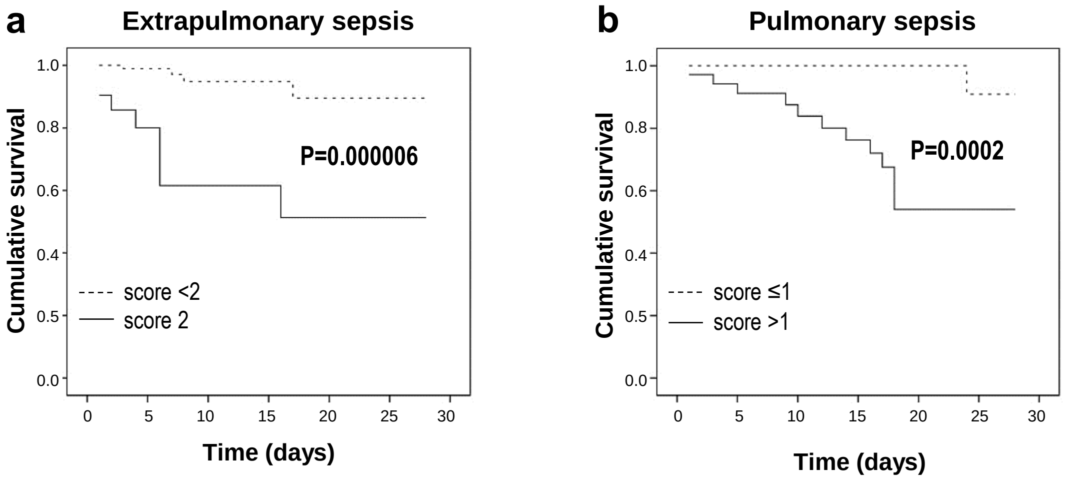

SOFA cut-off point: >8
PAI-1 cut-off point: ≥130pg/mL

NEOA cut-off point: >2
ICAM-1 cut-off point: ≥325ng/mL
AREG cut-off point: ≥53pg/mL

Supplementary Figure S3. Predictive value of biomarkers and clinical variables on cumulative ICU survival in septic patients.

Panels represent Kaplan-Meier survival curves for low and high scores of the best performing panels in patients with (a) extrapulmonary sepsis, and (b) pulmonary sepsis. The BMs and clinical variable that integrate the best performing panels are presented under the plots, including the cut-off values used for the scoring. Dotted lines represent the groups of patients with low total scores, and solid lines those with high total scores. ICU: Intensive Care Unit. BM: biomarker.

SUPPLEMENTARY RESULTS

Biomarkers levels

When analyzing the serum levels of IL-1RA and AREG in septic patients, a number of individuals had concentrations under the detection limit at the time of diagnosis (33.19% and 21.12% respectively).

In addition to the analysis of the BMs serum concentration, we measured BMs plasma levels at sepsis diagnosis in a different group of patients with IMV (48.33% with ARDS) (Supplementary Table S1). Compared to serum levels in patients with IMV, plasma concentrations of PAI-1, IL-18 and Ang-2 were significantly different (Supplementary Table S1). The proportion of individuals with IL-1RA levels under the detection limit was lower in plasma samples (13.79%).

SUPPLEMENTARY TABLES

Supplementary Table S1. Biomarkers levels in plasma *vs* serum in septic patients with invasive mechanical ventilation at sepsis diagnosis.

**Biomarker**	**Plasma (N=59)**	**Serum (N=151)**
RAGE (pg/mL)	1051.55 (530.63-2327.93)	1371.12 (645.05-2581.35)
PAI-1 (pg/mL)	23.66 (13.52-48.58) ***	82.93 (46.50-202.72)
SP-D (ng/mL)	5.98 (2.55-20.97)	6.73 (2.89-13.47)
IL-18 (pg/mL)	1140.88 (632.42-2128.59) ***	559.11 (303.20-1153.49)
Ang-2 (pg/mL)	8299.51 (3150.38-14537.66) **	3851.28 (1906.46-8105.40)
ICAM-1 (ng/mL)	344.45 (238.74-533.48)	383.21 (261.18-525.07)
CXCL16 (pg/mL)	5872.68 (3487.99-8202.56)	5020.01 (3400.91-7133.91)
AREG (pg/mL)	35.37 (23.09-81.13)	45.81 (26.55-101.06)
IL-1RA (pg/mL)	1563.06 (402.73-2812.32)	820.32 (19.55-10325.96)

Concentrations are given as median (Q1-Q3). N is the number of samples for each group.

**P<0.01 and ***P<0.001.

Supplementary Table S2. Comparison of serum biomarkers and clinical variables between septic patients with and without ARDS.

	ARDS sepsis (N=72)	Non-ARDS sepsis (N=160)	Non-ARDS sepsis with IMV (N=79)	Extrapulmonary sepsis with ARDS (N=22)	Extrapulmonary sepsis without ARDS (N=119)
**Biomarker**					
RAGE (pg/mL)	1907.77 (1008.21-3167.86)	843.45*** (461.54-1496.23)	1014.37## (467.99-2168.28)	1504.89 (732.46-3184.87)	810.07†† (436.91-1224.34)
PAI-1 (pg/mL)	68.18 (38.66-149.57)	74.61 (43.49-131.61)	90.60 (53.42-220.60)	85.37 (39.09-478.76)	84.87 (46.22-158.63)
SP-D (ng/mL)	7.65 (4.05-20.07)	5.64*** (1.92-9.55)	5.52# (2.08-11.56)	7.12 (4.37-9.05)	1.02† (1.74-8.85)
IL-18 (pg/mL)	684.82 (321.16-1452.97)	502.14* (299.82-937.13)	474.57 (300.86-1065.39)	649.31 (344.92-1070.66)	504.07 (299.54-978.75)
Ang-2 (pg/mL)	2879.48 (1447.94-6167.76)	5121.70*** (2692.76-10144.77)	4739.34## (2413.01-10541.61)	4033.96 (2569.77-7657.06)	5935.84 (3146.79-11228.66)
ICAM-1 (ng/mL)	378.39 (244.13-522.52)	343.12 (263.58-518.04)	357.84 (266.71-556.88)	469.04 (264.98-599.89)	347.43 (266.71-564.64)
CXCL16 (pg/mL)	5061.95 (3699.03-7143.02)	3787.83*** (2552.45-5910.11)	5020.01 (3195.95-7177.47)	5061.95 (4259.48-7783.05)	3784.59†† (2705.46-5714.32)
AREG (pg/mL)	39.38 (21.82-94.53)	36.19 (19.76-79.82)	46.21 (28.29-127.71)	37.45 (21.12-78.64)	39.13 (22.80-81.61)
IL-1RA (pg/mL)	414.59 (19.55-1809.27)	569.59 (19.55-3248.49)	1133.74 (19.55-12988.11)	802.93 (19.55-2744.73)	619.21 (19.55-4868.61)
**Clinical variable**					
PaO_2/FiO_2	135 (106.50-184.00)	266.35*** (183.73-386.18)	214.00### (151.70-294.30)	160.50 (124.00-193.73)	304.80††† (214.00-435.00)
APACHE II	18.00 (14.00-24.00)	17.00 (13.00-22.00)	19.00 (14.00-24.00)	21.00 (14.00-31.00)	18.00 (14.00-22.00)
SOFA	8.50 (6.00-10.80)	7.00** (5.00-9.75)	8.00 (6.00-11.00)	8.50 (7.00-11.25)	7.00† (5.00-9.00)
NEOA	2.00 (2.00-3.00)	2.00 (1.00-3.00)	3.00 (1.00-4.00)	2.00 (2.00-3.25)	3.00 (2.00-3.00)

Concentrations are given as median (Q1-Q3). N is the number of individuals in each group.

*P<0.05, **P<0.01 and ***P<0.001 for the comparison between ARDS patients and total non-ARDS patients.

#P<0.05, ##P<0.01 and ###P<0.001 for the comparison between ARDS patients and non-ARDS patients on IMV.

†P<0.05, ††P<0.01 and †††P<0.001 for the comparison between ARDS and non-ARDS in patients with extrapulmonary sepsis.

ARDS: Acute Respiratory Distress Syndrome. IMV: Invasive Mechanical Ventilation. APACHE II: Acute Physiology and Chronic Health Evaluation II. SOFA: Sequential Organ Failure Assessment. NEOA: Number of extrapulmonary organs affected.

Supplementary Table S3. Comparison of serum biomarkers and clinical variables between ICU survivors and non-survivors.

A.

	Total sepsis		Sepsis with IMV	
	ICU survivors (N= 195)	ICU non-survivors (N= 37)	ICU survivors (N= 116)	ICU non-survivors (N= 35)
**Biomarker**				
RAGE (pg/mL)	939.28 (533.67-1914.13)	1277.49 (564.29-3578.13)	1415.92 (654.02-2549.25)	1277.49 (566.13-2739.28)
PAI-1 (pg/mL)	68.17 (121.45-42.00)	129.35** (52.75-448.94)	75.58 (46.12-131.61)	129.35* (55.19-474.00)
SP-D (ng/mL)	5.51 (2.42-10.89)	6.84 (3.74-17.49)	6.52 (2.81-12.90)	6.84 (3.17-16.50)
IL-18 (pg/mL)	500.21 (298.45-935.79)	681.20* (458.56-1777.47)	487.79 (299.05-1042.87)	681.20* (375.37-1767.46)
Ang-2 (pg/mL)	4411.71 (2145.51-7872.58)	5950.23 (2352.10-9287.39)	3528.16 (1758.55-7403.03)	5595.17 (2341.23-9290.10)
ICAM-1 (ng/mL)	333.83 (248.05-507.52)	438.24* (336.55-581.53)	333.83 (239.99-497.76)	442.79* (357.84-582.76)
CXCL16 (pg/mL)	3968.94 (2672.32-5648.29)	6579.73*** (4432.83-10152.74)	4597.17 (3288.31-6409.59)	6579.73** (4438.86-10006.55)
AREG (pg/mL)	34.52 (12.79-71.67)	77.22*** (37.91-190.95)	28.05 (23.54-86.33)	78.00** (36.84-218.73)
IL-1RA (pg/mL)	368.60 (19.55-2516.48)	1581.70** (187.35-59562.44)	433.12 (19.55-2691.37)	1682.81** (235.82-60960.09)
**Clinical variable**				
PaO_2/FiO_2	228.00 (143.80-354.20)	163.80** (127.25-211.50)	183.55 (122.33-261.23)	159.80 (127.25-220.35)
APACHE II	17.00 (13.00-21.75)	23.50*** (15.00-29.00)	17.00 (13.00-22.00)	23.50*** (15.00-29.25)
SOFA	7.00 (5.00-9.00)	10.00** (6.50-12.50)	8.00 (6.00-10.00)	11.00* (6.00-13.00)
NOA	3.00 (2.00-4.00)	4.00** (3.00-5.00)	3.00 (2.00-4.00)	4.00 (3.00-5.00)

B.

	ARDS sepsis		Non-ARDS sepsis with IMV	
	ICU survivors (N=47)	ICU non-survivors (N=25)	ICU survivors (N=69)	ICU non-survivors (N=10)
**Biomarker**				
RAGE (pg/mL)	2222.26 (1245.97-3209.48)	1342.08 (853.36-2785.92)	1014.37 (502.32-2099.33)	815.03 (428.23-6034.87)
PAI-1 (pg/mL)	54.58 (35.22-114.10)	124.87** (61.98-483.52)	88.84 (53.27-206.26)	151.48 (49.06-851.73)
SP-D (ng/mL)	8.39 (4.50-17.71)	7.09 (2.92-20.81)	5.44 (2.04-11.01)	7.73 (3.60-12.87)
IL-18 (pg/mL)	644.16 (268.78-1064.24)	874.17* (602.16-2184.55)	452.55 (319.03-1049.64)	575.16 (109.11-1240.91)
Ang-2 (pg/mL)	2587.37 (986.63-4164.57)	5660.80*** (2473.49-9287.39)	4739.34 (2567.99-10653.82)	5714.63 (2009.71-9677.07)
ICAM-1 (ng/mL)	313.63 (216.07-495.73)	442.79** (356.31-620.85)	343.18 (263.95-560.11)	421.63 (243.09-504.54)
CXCL16 (pg/mL)	4491.22 (3288.31-5748.89)	6737.79** (4990.31-8929.03)	4790.64 (3254.91-6884.01)	7501.79 (2671.36-11186.13)
AREG (pg/mL)	33.88 (7.80-58.84)	82.95** (37.01-194.13)	45.81 (27.64-109.54)	79.21 (33.00-276.81)
IL-1RA (pg/mL)	131.53 (19.55-992.09)	1495.09*** (255.07-101235.30)	913.16 (19.55-7710.91)	1574.91 (19.55-79955.49)
**Clinical variable**				
PaO_2/FiO_2	128.80 (106.50-183.30)	146.30 (109.00-202.00)	224.00 (151.50-298.15)	196.65 (136.48-277.00)
APACHE II	16.00 (12.50-20.50)	24.00*** (21.00-33.00)	19.00 (13.75-23.25)	23.00 (13.75-28.25)
SOFA	8.00 (6.00-9.00)	11.00*** (8.50-13.50)	8.00 (6.00-10.00)	10.00 (3.00-12.00)
NEOA / NOA	2.00 (1.00-3.00)	3.00** (2.00-4.00)	3.00 (2.00-4.00)	4.00 (1.75-6.00)

C.

	Extrapulmonary sepsis		Pulmonary sepsis	
	**ICU survivors (N=127)**	**ICU non-survivors (N=14)**	**ICU survivors (N=65)**	**ICU non-survivors (N=21)**
**Biomarker**				
RAGE (pg/mL)	828.57 (461.48-1403.97)	853.36 (416.55-3251.20)	1838.89 (836.27-2930.49)	1811.86 (862.80-4466.41)
PAI-1 (pg/mL)	79.29 (46.22-131.89)	252.28* (71.04-649.14)	52.49 (35.77-85.95)	92.32* (44.86-318.12)
SP-D (ng/mL)	4.02 (1.76-8.78)	5.60 (3.89-11.52)	8.05 (4.42-16.55)	7.21 (3.79-7.21)
IL-18 (pg/mL)	495.18 (291.70-937.58)	777.69* (544.26-1934.13)	521.37 (302.46-816.26)	627.93 (217.48-1723.22)
Ang-2 (pg/mL)	5244.81 (2991.33-10541.61)	8133.36 (2631.96-9676.50)	2683.22 (940.38-4786.26)	4281.29* (1787.59-9287.39)
ICAM-1 (ng/mL)	355.58 (270.68-559.57)	448.03 (292.32-616.46)	290.52 (226.67-448.47)	427.78** (363.17-568.58)
CXCL16 (pg/mL)	3923.68 (2728.76-5648.29)	6552.03** (4780.40-10924.93)	3887.07 (2318.74-5522.11)	6579.73** (4372.50-9249.35)
AREG (pg/mL)	35.74 (20.05-71.34)	76.16** (50.61-116.59)	32.52 (7.80-78.71)	78.00** (32.48-336.57)
IL-1RA (pg/mL)	619.21 (19.55-3429.49)	6186.90* (398.73-151931.46)	132.79 (19.55-1169.58)	1682.81** (46.75-52846.02)
**Clinical variable**				
PaO_2/FiO_2	288.60 (195.00-427.50)	176.30** (133.30-271.23)	143.80 (100.00-186.65)	154.65 (98.25-206.00)
APACHE II	18.00 (13.00-22.00)	25.50** (14.00-30.75)	15.00 (9.50-19.00)	21.50** (15.00-27.50)
SOFA	7.00 (5.00-9.00)	10.00** (8.75-13.25)	7.00 (6.00-9.00)	10.00 (4.50-12.50)
NOA	3.00 (2.00-4.00)	4.00* (3.00-6.00)	3.00 (2.00-4.00)	3.00 (3.00-4.00)

Concentrations are given as median (Q1-Q3). N represents the number of individuals in each group.

*P<0.05, **P<0.01 and ***P<0.001 for the comparison between ICU survivors and ICU non-survivors in each group.

ICU: Intensive Care Unit. IMV: Invasive Mechanical Ventilation. APACHE II: Acute Physiology and Chronic Health Evaluation II. SOFA: Sequential Organ Failure Assessment. NOA: Number of organs affected. ARDS: Acute Respiratory Distress Syndrome. NEOA: Number of extrapulmonary organs affected.
